# Citizen participation in healthcare: a field perspective: Rob Timans

**DOI:** 10.1093/eurpub/ckac131.545

**Published:** 2022-10-25

**Authors:** R Timans, J de Jong, A Brabers, M Horsselenberg, L Damen

**Affiliations:** 1 ZOS, Nivel, Utrecht, Netherlands; 2 Faculty of Health, Medicine and Life Sciences, Maastricht University, Maastricht, Netherlands

## Abstract

**Background:**

Many nations are faced with challenges regarding the sustainability of their healthcare systems. Rising costs and limited financial and human resources mean that difficult decisions regarding the provision of healthcare are imminent. Which care should be provided, when and where? These decisions will affect all current and future users of healthcare. This has given rise to calls from policy makers and others to include citizens in the decision making process. The question is how this can be done. What are viable ways of citizen participation in healthcare? What are critical issues to take into account? And how do citizens themselves want to participate?

**Methods:**

We use results from a literature review, six interviews with organizations in the field and the results of two citizen platforms to map the challenges and opportunities for citizen participation in healthcare. We study the case of the Netherlands, a country with a long and singular tradition of participatory policies.

**Results:**

Preliminary findings indicate there are six key decision variables that are instrumental in shaping citizen participation in the field of healthcare. Among those are the kinds of knowledge that participants possess and the valuation of these kinds of knowledge by agents in the field of healthcare.

**Conclusions:**

We interpret the results using a Bourdieusian conceptual framework, which emphasizes the contextual framing of participation. Citizen participation can be understood as an intervention in the field of healthcare. Participation is structured by the different forms of knowledge of participants as capitals. This highlights power differences that must be understood in order to design fruitful participatory processes.

**Key messages:**

• Citizen participation in healthcare understood as an intervention in the field of healthcare.

• Understanding power differences is crucial for designing and implementing this intervention.

